# Applications of Multimodal Imaging in Central Serous Chorioretinopathy Evaluation

**DOI:** 10.1155/2021/9929864

**Published:** 2021-07-24

**Authors:** Mary Ho, Gabriel Li, Andrew Mak, Danny Ng, Lawrence Iu, Frank Lai

**Affiliations:** ^1^Department of Ophthalmology and Visual Sciences, The Chinese University of Hong Kong, Shatin, Hong Kong; ^2^Department of Ophthalmology and Visual Sciences, Prince of Wales Hospital, Shatin, Hong Kong; ^3^Department of Ophthalmology and Visual Sciences, Hong Kong Eye Hospital, Kowloon, Hong Kong; ^4^Department of Ophthalmology, Caritas Medical Centre, Sham Shui Po, Hong Kong

## Abstract

Central serous chorioretinopathy (CSCR) is a macular disease characterized by serous retinal detachment commonly involving the macular region. CSCR has a wide spectrum of clinical presentations. Although a significant proportion of CSCR cases are self-limiting, patients can suffer from persistent or recurrent disease, sometimes complicated with choroidal neovascularization, resulting in permanent visual loss. Multimodal imaging, including fluorescein angiography, indocyanine green angiography, fundus autofluorescence, and optical coherence tomography, has advanced the diagnosis and classification of CSCR cases. Evolution of new imaging techniques including optical coherence tomography angiography, wide-field imaging, and en face reconstruction imaging has also contributed to better understandings of the pathophysiology of CSCR. This review article summarizes the features of multimodal imaging for CSCR and discusses the application of such features in evaluating the disease.

## 1. Introduction

Central serous chorioretinopathy (CSCR) is a common retinal disorder. Eyes suffering from CSCR demonstrate focal or multifocal areas of neurosensory retinal detachment and different degrees of retinal pigment epithelium (RPE) cell loss, coupled with evidence of a thickened or dysfunctional choroid [1, 2]. CSCR is generally classified as either acute or chronic based on the duration of serous retinal detachment; in eyes with acute CSCR, detachment has occurred within 3 to 6 months, whereas in eyes with chronic CSCR, subretinal fluid (SRF) has persisted for more than 3 to 6 months [3, 4]. However, this classification may not thoroughly describe or provide prognostic values for the disease within a wide spectrum of CSCR phenotypes [5].

Adopting information from multimodal imaging is hence crucial to evaluating CSCR. Multimodal imaging techniques including fluorescein angiography (FA), indocyanine angiography (ICGA), fundus autofluorescence (FAF), and optical coherence tomography (OCT) are important in guiding CSCR diagnosis and providing prognostic information on the visual outcome. Traditional imaging techniques including FA and ICGA have irreplaceable roles in CSCR diagnosis. FA identifies focal leaks through RPE defects and reveals serous RPE detachment and other RPE alterations as well. ICGA reveals abnormal choroidal vasculature including delays in choroidal filling, dilated choroidal vessels, and areas of choroidal hyperfluorescence [6, 7]. Among more recent techniques, OCT is commonly used to evaluate CSCR cases through confirming any serous retinal detachment, as well as providing information on the presence of outer retinal abnormalities, serous RPE detachments, subretinal fibrin, and increased choroidal thickness [6]. FAF provides information on RPE alterations and the extent of the disease [1]. Lastly, optical coherence tomography angiography (OCTA) is a new tool used to detect the presence of choroidal neovascularization (CNV); when combined with features on OCT scans, it has a sensitivity and specificity comparable to FA. All these multimodal imaging techniques have improved our understanding of CSCR disease severity and chronicity [2].

This review article summarizes the key features of multimodal imaging techniques for CSCR and provides an overview on the application of different imaging modalities in evaluating this disease with a wide spectrum of presentations.

## 2. Methods

The PubMed, EMBASE, and Web of Science databases were searched for articles published between 2000 and 2020 using the keyword terms “central serous chorioretinopathy” OR “pachychoroid” OR “neovascularization” OR “neovascular” OR “fluorescein angiography” OR “indocyanine angiography” OR “fundus autofluorescence” OR “optical coherence tomography” OR “optical coherence tomography angiography.” The search did not apply a language filter. After excluding irrelevant or duplicate articles, the references for the included articles were reviewed to identify additional relevant studies. The current review excludes statements, editorials, letters to the editor, and reviews; however, case series and case reports of particular significance are mentioned in relevant sections.

### 2.1. Fluorescein Angiography

CSCR was first described by Albrecht von Graefe in 1866 as serous retinal detachment, either with or without serous pigment epithelial detachment (PED). Although CSCR diagnoses can be made clinically and with noninvasive investigations such as OCT, findings from FA and ICGA can assist CSCR treatment and provides insights into the choroidal changes associated with CSCR.

In acute CSCR, typically one—sometimes multiple—fluorescein leakage site is present at the level of the RPE, leading to subretinal accumulation of dye [1]. Three different patterns of leakage have been described, including the smokestack pattern, the inkblot, and the minimally enlarging spot [2]. The smokestack pattern is characterized by ascending hyperfluorescence followed by lateral diffusion, producing a mushroom-like image above the leaking point. The more common inkblot leakage pattern is characterized by a progressive and uniform expansion of a circular hyperfluorescence arising from a central pinpoint. A study of 86 CSCR patients observed the inkblot pattern in 53% of eyes [1]. In contrast, the smokestack pattern is predominantly associated with an acute early form of CSCR, which was observed in 14% of subjects in a study of 479 acute, recurrent, and chronic CSCR cases [3]. In the mid and late phases of FA, the pooling of dye occurs in areas of serous retinal detachment, giving a diffuse and circular spreading of hyperfluorescence.

In chronic CSCR, diffuse RPE damage with broad areas of serous retinal damage is often present. FA therefore shows multiple indistinct leakages or the diffuse oozing of dye, usually in the form of a descending tract, resulting in patches of granular or mottled hyperfluorescence in the mid and late phases [4]. Lastly, in eyes with resolved CSCR or fellow eyes, areas of early hyperfluorescence are present due to increased transmission from window defects that are caused by localized RPE atrophy [5].

### 2.2. Indocyanine Green Angiography

**Figure 1:**
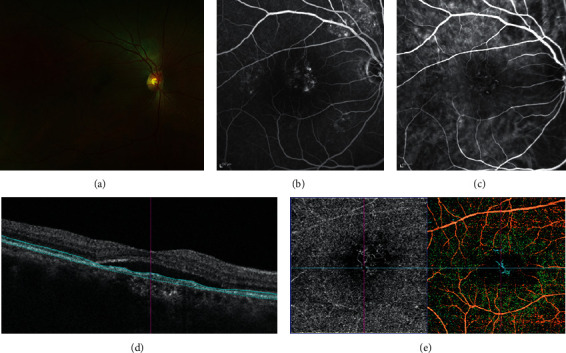
Diagnosis of polypoidal choroidal vasculopathy (PCV) in a case of central serous chorioretinopathy. Subretinal fluid is demonstrated with fundus photograph (a). Multifocal leakage is seen in fluorescein angiography (b). Polypoidal lesions and branching vascular network (BVN) are seen in indocyanine green angiography (c). Optical coherence tomography (OCT) does not show specific feature of PCV (d). BVN, but not polypoidal lesions, can be seen in OCT angiography (e).

ICGA makes use of the high molecular weight and amphiphilic nature of indocyanine, which allows for the staining of intravascular content, vascular walls, and extravascular structures such as the choroidal stroma. ICGA findings in CSCR can be categorized into early, mid, and late phase findings. In early phase, there is a delay in filling of choroidal arteries and choriocapillaris, resulting in areas of hypofluorescence that persists into the mid and late phases [6]. In the mid-phase, focal choroidal hyperfluorescence surrounding the leakage point occurs due to choroidal vascular hyperpermeability [7]. Dilated large choroidal veins are also seen in areas of atrophic or elevated RPE. These mid-phase hyperfluorescent areas develop into persistent, washout, or centrifugally displaced hyperfluorescence in the late phase [8]. In addition, choroidal vessels exhibit transient hyperpermeability, shown as multiple punctate hyperfluorescent spots, increasing in severity in the mid-phase and fading in the late phase. These spots are also observed in unaffected areas of the retina and in contralateral eyes, suggesting that choroidal hyperpermeability plays an essential role in the pathogenesis of CSCR [8, 9]. Lastly, ICGA also helps to identify CNV that complicates CSCR. In these eyes, there is increasing hyperfluorescence from the mid to the late phase, showing an ill-defined, late-staining plaque that confirms the presence of occult CNV [10]. Associated polypoidal choroidal vasculopathy (PCV) can also be identified with ICGA (Figure 1). Branching vascular network and polypoidal lesions are characteristic ICGA features of PCV [8]. In both EVEREST criteria [8] and Japanese Study Group guideline [9], ICGA is the gold-standard investigation to diagnose PCV. It is also important to identify CSCR complicated with PCV to decide the need of anti-VEGF therapy, in addition to photodynamic therapy [10].

ICGA has been used to guide the treatment of CSCR with photodynamic therapy for nearly two decades [11]. The area of choroidal dilation and hyperpermeability in ICGA is used to delimit the area to treat [12]. In the PLACE trial, ICGA has been used to guide the half-dose photodynamic therapy and subthreshold micropulse laser [13]. Half-dose photodynamic therapy can provide a higher rate of complete subretinal fluid resolution and functional improvement than subthreshold micropulse laser [13]. Besides, the absence of hyperfluorescent abnormalities in ICGA is a potential predictor of poor treatment response after photodynamic therapy [14, 15].

### 2.3. Optical Coherence Tomography

In the past decade, spectral domain OCT (SD-OCT) has been the imaging modality of choice for evaluating and following up CSCR. SD-OCT provides reliable and reproducible noninvasive imaging of the retina. When supplemented with enhanced depth imaging (EDI-OCT) or swept-source OCT (SS-OCT), deeper structures such as the choroid and choroid-scleral interface can be visualized, and parameters such as subfoveal choroidal thickness (SFCT) can be evaluated.

#### 2.3.1. General Features

CSCR has been increasingly recognized as an entity of the pachychoroid spectrum [16]. A thickened choroid is an important feature of this disease spectrum, which is a group of diseases driven by choroidal dysfunction. The exact definition of a thick choroid is debated, with values ranging from 300 *μ*m [17] to 395 *μ*m [18] being proposed as the threshold of normal SFCT; nevertheless, some argue that there is no definitive qualitative threshold given the many parameters that can affect choroidal thickness [19]. Compared to normal healthy control subjects, the thickening of the choroid is seen in eyes with CSCR [20, 21] and the asymptomatic fellow eyes of CSCR patients [22].

Apart from a thickened choroid, the attenuation of the inner choroidal layers, especially the choriocapillaris, is observed in an area overlying a thickened Haller's layer with dilated vessels [23]. In addition, OCT can easily detect SRF accumulation, a hallmark of CSC. Lastly, PED, a common feature in CSCR, can occur within or outside the area of neurosensory retinal detachment [24]. OCT has been evaluated to diagnose PCV without ICGA. Subretinal pigment epithelium ring-like lesion, en face OCT complex RPE elevation, and sharp-peaked PED can achieve an area under the receiver operating characteristic curve of 0.90 in diagnosing PCV [25].

#### 2.3.2. Acute CSCR

Aside from SRF accumulation, retinal morphology usually remains unchanged in acute CSCR cases. Areas of serous retinal detachment sometimes present elongations of the photoreceptor outer segment, which is postulated to be due to the lack of phagocytosis by the RPE [26].

For acute CSCR cases, focal RPE defects and associated PED have been reported, being localized to the leakage site detected through FA [27]. Small RPE bump [28], fibrinous exudates in the subretinal space [27], and the sagging or dipping of the posterior layer of the neurosensory retina above the leakage sites [27, 28] have all been reported to coincide with the leakage site.

#### 2.3.3. Chronic CSCR

Morphological changes in the retina have been described as accompanying the persistence of SRF in chronic CSCR. The thinning of the outer nuclear layer (ONL) is noted during the early onset of the disease, and continuous thinning is noted with the persistence of SRF [29]. Cystoid changes appearing as intraretinal hyporeflective spaces, a process known as cystoid macular degeneration, are also commonly seen. These changes remain after the resolution of the SRF [30].

As in acute cases, intraretinal and subretinal hyperreflective dots have been observed in chronic CSCR [31]. These deposits may result from the accumulation of proteins or macrophages within the phagocytized photoreceptor outer segments. Intraretinal deposits are seen during the early onset of the disease, with progressive migration into deeper layers occurring during disease evolution [32]. Notably, these hyperreflective dots appear to be colocalized with the hyperautofluorescent dots composing the granular hyperautofluorescence seen during the resolution of SRF [33]. These OCT hyperreflective deposits will resolve slowly with the resolution of SRF. Yet as the SRF persists in chronic cases, these hyperreflective dots will increase in number and thus be more commonly seen in chronic cases [34].

**Figure 2:**
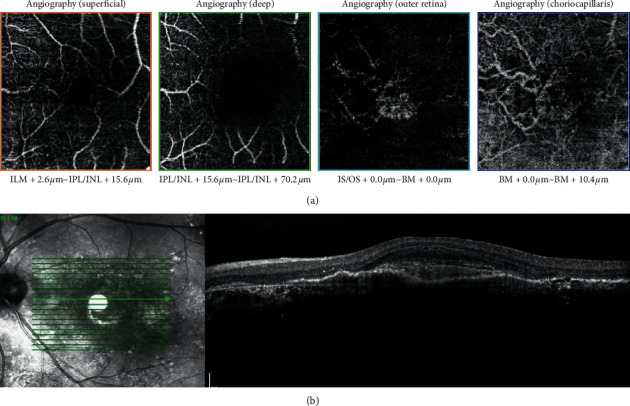
Association of choroidal neovascularization with flat irregular pigment epithelial detachment (FIPED). (a) Optical coherence tomography (OCT) angiography at the outer retina layer and the choriocapillaris level shows evidence of choroidal neovascularization. (b) Correlation with the OCT scan of the same patient, which shows flat irregular pigment epithelial detachment, fibrinous exudates, and evidence of shallow neurosensory retinal layer detachment.

PED is a feature common to both acute and chronic CSCR. In particular, flat irregular PED (FIPED) is usually found in the chronic form (Figure 2) [35–37]. An OCT scan of FIPED can visualize two hyperreflective lines, comprising an undulated RPE line and a hyperreflective straight line representing Bruch's membrane; it is thus also known as a double-layer sign [37]. The relationship of FIPED with CNV will be discussed in the section on OCTA.

## 3. OCT Prognostic Factors

### 3.1. Hyperreflective Dots

A greater number of intraretinal hyperreflective dots at baseline correlates with a longer duration of SRF resolution [38]. The number of subretinal hyperreflective dots is associated with recurrences of CSCR [38, 39]. The presence of both types of dots in resolved CSC is associated with poor visual acuity [40].

### 3.2. Cystoid Macular Degeneration

Cystoid macular degeneration [41] is a prognostic factor of poor visual outcomes. It must be differentiated from cystoid macular edema, which is accompanied by active angiographic leakage [30]. However, eyes with cystoid macular degeneration that spares the foveal center may have preserved vision [42].

### 3.3. Outer Retinal Changes

Several outer retinal morphological changes are associated with poor visual outcomes in CSCR. The ellipsoid zone (EZ), formerly known as inner segment/outer segment junction, was renamed after the anatomical correlation was discovered between this hyperreflective line and the photoreceptor inner segment ellipsoid. Being densely packed with mitochondria, in a disease state, the integrity of the EZ is often correlated with visual function as it reflects the metabolism of the photoreceptors [43]. Various reports have associated EZ disruption with poor visual acuity [40, 41, 44–46]. Such negative impacts on visual acuity are not limited to the mere presence of EZ disruption; the length [40] and the extent [47] of the disruption also affect visual acuity. Aside from these prognostic implications for visual acuity, the residual EZ in the central macula of resolved CSC eyes predicts microperimetric retinal sensitivity [46, 47]. EZ disruption at baseline also correlates with a delayed resolution of SRF [48].

Besides EZ disruption, other prognostic factors associated with poor visual acuity include the disruption of the external limiting membrane (ELM), another hyperreflective line of the outer retina [40, 41], in addition to ONL thinning [44, 45].

### 3.4. Subfoveal Choroidal Thickness

The effect of SFCT on visual outcome has been subject to debate. A greater SFCT at baseline has been shown to correlate with a longer duration of SRF persistence [4] and a higher chance of requiring treatment [49]. A higher recurrence rate is also implicated in patients with a greater SFCT [39]. While SFCT does not differ significantly between typical acute and chronic cases, greater SFCT has been observed with multifocal CSCR [50]. However, caution must be exercised when considering SFCT as a prognostic factor. SFCT is usually measured manually, and the identification of the choroidal extent can be difficult. Significant inter- and intraobserver variability also exists for choroidal thickness measurement, especially in disease state such as CSCR [51].

### 3.5. Fundus Autofluorescence

**Figure 3:**
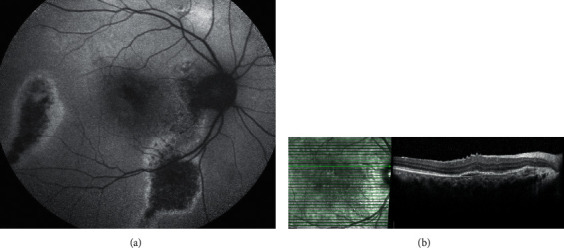
Fundus autofluorescence (FAF) reveals a much larger extent of central serous chorioretinopathy involvement than optical coherence tomography (OCT) alone. (a) FAF shows evidence of water tract signs and areas of confluent hypoautofluorescence (hypo-AF) changes nasal and temporal to the macula, surrounded by edges of hyperautofluorescence changes. The macular area also shows evidence of granular hypo-AF changes, signifying an impaired visual function in this case. (b) OCT scan of the same patient shows resolved subretinal fluid and evidence of retinal pigment epithelium alterations.

FAF is a noninvasive imaging technique that indirectly evaluates the functions of the outer retina and the RPE. The intensity and spatial distribution of FAF have been described in various macular diseases including CSCR [52–55]. FAF is an in vivo marker of the photoreceptor and RPE function. Lipofuscin, which has autofluorescent properties, accumulates within the RPE in the presence of the incomplete phagocytosis of photoreceptor outer segments [56]. FAF can thus provide indirect information on the metabolic activity of the RPE, as lipofuscin levels are influenced by turnover rates for the photoreceptor outer segments [52, 56, 57]. In view of this, reports have considered hypoautofluorescence involving the fovea as a marker of impaired visual function (Figure 3) [58–60]. There are two types of FAF imaging techniques, namely, short-wave FAF (SW-FAF) and near-infrared FAF (NIR-FAF). The former originates from the lipofuscin pigment of the RPE, while the latter originates from the melanin pigment of both the choroid and the RPE [61]. Of the two modalities, SW-FAF is more commonly used [58].

Different autofluorescence (AF) patterns have been described in CSCR. In acute CSCR, areas of subretinal detachment show hypo-AF due to AF masking originating from the RPE by SRF, as well as early elongation of photoreceptor outer segments [62]. Moreover, Spaide et al. observed diffuse homogenous hyper-AF patterns in acute CSCR, both with and without the presence of hyper-AF punctate dots [54]. Similarly, NIR-FAF imaging also shows granular hyper-AF for acute CSCR, with FAF patterns at the leakage site of CSCR being previously reported [53]. In acute CSCR, focal areas of hypo-AF correspond to the site of focal RPE leakage [52]. Lacono et al. reported similar appearances of well-defined hypo-AF at the leakage site [33]. In some cases, such hypo-AF may be due to focal defects of the RPE found within the PED and correspond precisely to the leakage point detected using FA [27, 52]. Nevertheless, reports have shown that not all eyes with CSCR have hypo-AF features at leakage point [63].

In general, a gradual nature of change in FAF patterns was observed in eyes with chronic CSCR. In their case series evaluating FAF patterns in CSCR patients, Zola et al. revealed the earliest change in chronic CSCR to be diffuse hyper-AF, occurring approximately 4 months after disease onset; granular hypo-AF or diffuse hypo-AF was gradually observed with longer disease duration [64]. Of the different patterns, diffuse homogenous hyper-AF usually indicates the presence of SRF or reactivation of disease, while punctate or dot hyper-AF spots represent chronicity related to the accumulation of lipofuscin in photoreceptor outer segments [35, 64–66]. With prolonged disease duration, granular and confluent hypo-AF was observed in around 25% of cases at 24–36 months [64]. Hypo-AF is associated with RPE cell loss and thinning of the posterior surface of the detached retina. In the presence of granular hypo-AF, a subtotal or subconfluent loss of cells is a possible explanation, while homogenous or confluent hypo-AF is observed in the presence of confluent cell loss. Hence, confluent hypo-AF is a poor visual prognostic factor in CSCR [62, 67]. Studies have shown a good correlation between FAF patterns and functional outcome as measured by microperimetry and visual acuity; FAF is hence a valuable tool for estimating the functional impairments of CSCR cases [68].

### 3.6. Choroidal Imaging Findings

#### 3.6.1. Choroidal Thickness

CSCR has recently been recognized as a member of the pachychoroid disease spectrum. Choroidal thickness was reported to have increased in both the eye with CSCR and in the fellow eye of CSCR patients [20, 69, 70]. This increased thickness is postulated to be related to the dilation of large choroidal vessels with both eyes being involved [16]. Many studies have presented evidence to support the bilateralism of CSCR [70, 71]. In their series of 173 patients, Rijssen et al. revealed no statistically significant differences in choroidal thickness between the affected eye and the fellow eye in untreated chronic CSCR [70]. In their cohort study evaluating choroidal layer thickness in CSCR, Chhablani et al. showed that SFCT, along with the widths of medium and large choroidal vessels, had increased in eyes with acute CSCR compared to healthy control eyes [72]; they reported the SFCT was to be 360 *μ*m in eyes with acute CSCR, 338 *μ*m in eyes with chronic CSCR, and 277 *μ*m in normal eyes. Moreover, investigations of the different choroidal layers for resolved CSCR observed changes in thickness for both the subfoveal choroidal thickness and Haller's layer, while the thickness of Sattler's layer remained unchanged [73]. In view of the current evidence, choroidal interstitial edema has been postulated to play a role in the thickened choroid observed for CSCR. Although CSCR is now considered as a pachychoroid spectrum disorder, it does not require thickened choroid for diagnosis as different factors including age, refractive status, and axial length can alter choroidal thickness [1].

Studies have reported that the choroidal thickness of eyes with CSCR decreases after photodynamic therapy (PDT). PDT is postulated to induce vascular endothelial damage and thrombus formation, causing short-term choriocapillary occlusion and long-term choroidal vascular remodeling. These changes can normalize the width of dilated, congested choroidal vessels [74, 75]. In their evaluation of choroidal thickness in eyes with CSCR treated by either laser photocoagulation or half-dose PDT, Maruko et al. found choroidal thickness to be reduced only in the PDT group [76]. Several subsequent studies have also shown a decrease in choroidal thickness for eyes with CSCR after half-fluence PDT [77, 78]. In addition, Kim et al. reported that the rate of reduction for the thickness of subfoveal choroidal layer was highly predictive of CSCR recurrence, with changes occurring as early as 1 month after treatment [79]. Changes in SFCT may hence reflect treatment efficacy and help predict recurrence rate.

#### 3.6.2. Choroidal Vascularity Index

Different studies have evaluated the choroidal vascularity index (CVI) as an objective parameter of choroid evaluation for CSCR cases. Vascular alternations in the choroid, which mostly comprises blood vessels and choroidal interstitial stroma, are believed to be involved in the pathogenesis of CSCR. CVI—defined as the area of the vessels divided by the total choroidal stromal area—thus emerges as an imaging biomarker for CSCR status. Agrawal et al. reported increased CVI from the EDI-OCT scans of eyes with active disease compared to eyes with resolved disease, normal fellow eyes, and age-matched healthy eyes. CVI parameters can be calculated based on enhanced depth imaging, and such changes can be adopted as evaluative indicators of CSCR status, given that a change in CVI can indicate either disease resolution or a positive treatment response [80, 81].

#### 3.6.3. En Face Images of the Choroid

The combination of SS-OCT imaging and transverse confocal analysis produces transverse images, en face images of the retina and the choroid at specific depths [82]. This en face SS-OCT imaging shows detailed patterns of the choroidal vasculature in eyes with CSCR, revealing inner choroidal layer thinning at the choriocapillaris in involved areas [32] and outer choroidal vessels dilatation [33]. In their evaluation of the choroidal structure using en face images, Ferrara et al. revealed that 53% of cases had focally enlarged vessels at the choriocapillaris layer and at Sattler's layer, while 80% showed diffuse dilation at Haller's layer [83]. Similarly, in their evaluation of CVI values for CSCR, Wong et al. observed dilatation at Haller's layer, as well as attenuation of both the Sattler and choriocapillaris layers, for both the acute and chronic states of CSCR [84]. These findings support the hypothesis that the dilated Haller's layer compresses the smaller inner choroidal vessels, resulting in the leakage and accumulation of serous fluid due to vascular insufficiency in the inner layer. En face OCT images also provide a glimpse into the morphological differences in vessel arrangement in Haller's layer; they reveal a “reticular pattern” in both acute and chronic CSCR eyes that contrasts with the “herringbone pattern” of healthy eyes [85–87]. En face OCT is thus a helpful tool for visualizing and evaluating the choroidal vessels.

#### 3.6.4. Vascular Reactivity Changes

Altered vascular reactivity responses at the choroid may be a plausible cause for the development of CSC R. Certain studies have shown that hypertensive patients have a higher risk of CSCR [60]. Others have shown that altered choroidal vascular response and increased vascular density in CSCR patients under stress conditions lead to increased blood pressure [88]. The physiological mechanisms that regulate the homeostasis of choroidal circulation may thus be dysfunctional in CSCR patients, which involves vascular endothelial autoregulation dysfunction [89], myogenic regulatory mechanism failure [90], and sympathetic overreactions [91].

### 3.7. Optical Coherence Tomography Angiography

#### 3.7.1. Improved Understanding of Pathogenesis Using OCTA Analysis

OCTA is a noninvasive investigative method that allows for the qualitative and quantitative assessment of vascular structures in the retina and the choroid [92], thus enabling researchers to investigate the potential pathogenesis of CSCR. Swept-source OCTA has already demonstrated that eyes with CSCR have a larger vascular flow area in the choroid compared to healthy control eyes, supporting the involvement of choroidal circulation in CSCR pathogenesis [92]. These regions of dilated choroidal vessels closely correspond to the regions where SRF and outer retinal changes are observed [93]. The coarse granulated high reflective areas found in CSCR patients through OCTA may also correspond with the hyperpermeability area found through ICGA [94]. In addition, retinal vascular alterations were observed in CSCR eyes, including vascular rarefaction, the enlargement of the foveal avascular zone, and telangiectasias [95]. Evidence has shown that a rapid increase in blood pressure and ocular perfusion pressure can alter retinal blood flow in CSCR eyes, with Piccolino et al. suggesting that an abnormal autoregulatory mechanism modulates retinal microcirculation and choroidal blood flow in such cases [96]. Out of the different choroidal layers, the choriocapillaris is likely the most vulnerable to variations of systemic hemodynamics [88]. In their evaluation of the vascular characteristics of the choriocapillaris in normal and pachychoroid eyes, Spaide et al. found that in pachychoroid eyes with manifested disease, the choriocapillaris vessel diameter was larger compared to normal eyes or pachychoroid eyes without manifested disease [97]. OCTA can also detect choriocapillaris hypoperfusion as a subclinical change preceding CSCR and other pachychoroid spectrum disorders [98]. In addition, studies using OCTA analysis showed zones of reduced choriocapillaris flow in CSCR patients, both with or without surrounding hyperperfusion, potentially suggesting that inner choroidal ischaemia leads to SRF leakage [99–101].

#### 3.7.2. Posttreatment Changes in Retinal and Choroidal Vasculatures

Aside from the pathogenesis of CSCR, OCTA allows clinicians to better understand the mechanism of different treatment modalities, as it provides information on the blood flow of different choroidal layers, especially the choriocapillaris layer. PDT, the most commonly adopted treatment method for CSCR eyes, mainly acts on larger choroidal vessels while sparing choriocapillaris blood flow [102]. In a randomized trial comparing half-dose PDT and micropulse laser treatments for CSCR, PDT had a stronger effect of promoting the recovery of choriocapillaris perfusion [103]. Researchers have also shown the anatomical benefits of PDT on CSCR eyes using OCTA analysis, which showed improved choriocapillaris perfusion signals and improved SFCT [104]. Nevertheless, abnormal choriocapillaris flow attenuation may still remain in completely resolved CSCR eyes, even after half-dose PDT treatment [105]. OCTA also improves clinical understandings of treatment-resistant CSCR eyes. Cennamo et al. investigated the vascular changes in the retinal and choriocapillaris networks among responders and nonresponders to low-fluence PDT. The responder group showed significant increases in vessel density at the deep capillary plexus and the choriocapillaris after treatment, while no changes were observed in the nonresponder group. To conclude, OCTA has the potential to become a new biomarker for evaluating treatment efficacy on CSCR eyes [106]. Abnormal choriocapillary flow patterns can be associated with higher recurrence rates in resolved CSCR eyes [107].

#### 3.7.3. Relationship between Choroidal Neovascularization and Central Serous Chorioretinopathy

**Figure 4:**
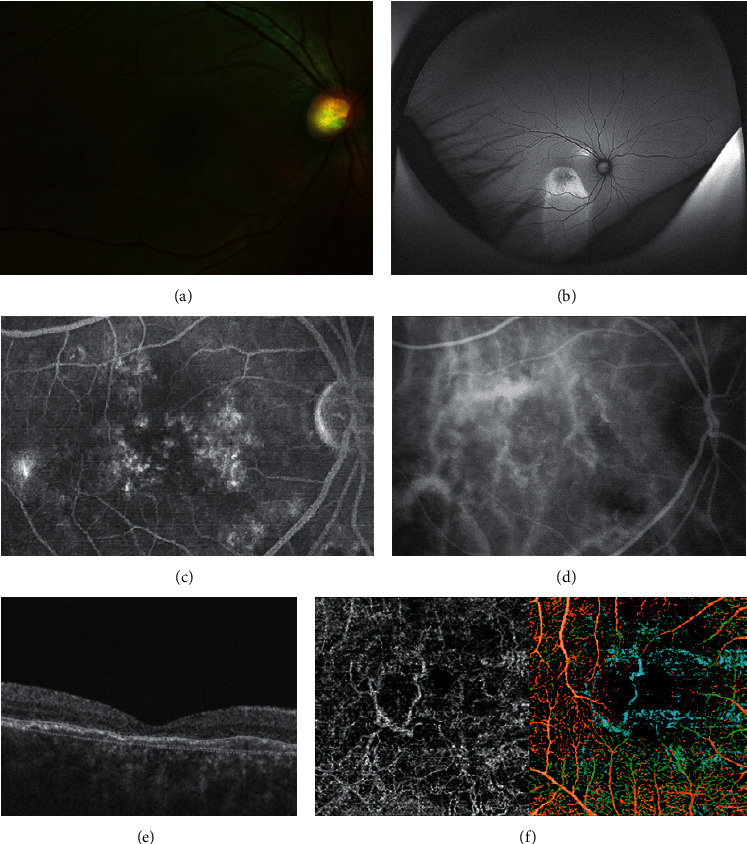
Application of multimodal imaging in assessment of central serous chorioretinopathy. (a) Fundus photograph reveals change in the retinal pigment epithelium (RPE) layer. (b) The extent of the RPE layer change is more prominent with the fundus autofluorescence. (c) Fluorescein angiography demonstrates staining pattern of hyperfluorescence without active leakage. (d) Underlying choroidal hyperfluorescence is shown with indocyanine green angiography. (e) Although no subretinal fluid or pigment epithelial detachment is found, choroidal neovascularization is noted at the choriocapillaris layer (f), which is colored blue in the composite optical coherence tomography angiography.

OCTA is sensitive enough to identify the various features of the choroidal neovascular network in CSCR eyes, which have been underreported from other imaging modalities such as FA and ICGA (Figure 4) [108, 109]. Using OCTA, the choroidal neovascular network was found to have either a loop-like or a tree-like structure. OCTA also revealed similar features in CSCR eyes with PED, as half of such eyes were found to have neovascular networks [110]. OCTA has the advantages of high sensitivity and specificity; studies have reported 71–86% sensitivity and 81–96% specificity using OCTA to detect CNV in CSCR eyes [111, 112]. OCTA can hence facilitate CSCR diagnosis when evidence for CNV is inconclusive following FA or ICGA.

OCTA scans can also delineate other features associated with the choroidal neovascular membrane. Cennamo et al. found that CSCR patients complicated by CNV had a lower choriocapillary vascular density that might be responsible for the development of CNV [113]. Likewise, Sahoo et al. reported that choroidal neovascular networks were found in 45% of eyes with cystoid macular degeneration, as well as that a persistent and shallower SRF might suggest the presence of a choroidal neovascular network [114]. Moreover, recent evidence shows that flat irregular PED (FIPED) can be a risk factor indicating the presence of CNV (Figure 2) [111, 115]. Using OCTA scans, Liu et al. showed that CNV was found in 7% of CSCR patients, and that among this group, all eyes with CNV had concurrent FIPED [116]. In their evaluation of the morphology of PEDs in CSCR eyes, Hwang et al. analyzed the natural course of CSCR eyes with FIPED and focal PED using OCTA, revealing that the former exhibited more CNV and lower rates of complete resolution of SRF. Yet eyes with FIPED are responsive to intravitreal bevacizumab injections, supporting the theory that FIPED is a form of pachychoroid neovasculopathy [117]. In addition, Sacconi et al. showed that the vascular density of CNV-complicated chronic CSCR did not change after PDT and intravitreal aflibercept injections, suggesting that arteriogenesis is the major driving force behind CNV [118]. Although vascular density remains unchanged and the mean area of CNV sustains following half-dose PDT treatment, unless there is prominent CNV activity, the treatment can still achieve improvements in visual acuity and SRF resolution at 6 months [119].

Because of the higher detection rates through OCTA, CNV was shown to be common in CSCR eyes after PDT. Wu et al. reported that CNV could be diagnosed in 46% of CSCR eyes after PDT. The risk factors associated with CNV development include older age, larger PDT spot size, and lesser SFCT [120]. Wider PED and the recurrence of CSCR are also postulated to be risk factors of secondary CNV development [121]. Nonetheless, Chen et al. reported CSCR eyes with CNV identified posttreatment could have a stable clinical course of up to 3 years, with SRF only being observed in 17% of eyes after 3 years [122]. On the other hand, given that the treatment response of CNV to anti-VEGF therapy can be variable and often incomplete, OCTA can help to identify potential better responders. In their investigation of the potential predictors of treatment response to anti-VEGF therapy for secondary CNV in CSCR, Romdhane et al. found that CNV with greater size and larger flow area detected using OCTA was associated with a better chance of complete response to anti-VEGF therapy [123]. OCTA can help identify subclinical cases of CNV and predict complicated case. For example, in a study of 40 eyes, OCTA could identify one-fourth of fellow eyes harboring CNV that were not detected using conventional imaging [124]. Subclinical CNV carries a clinical significance, as a conversion to exudative form can be seen in one-third of eyes with subclinical CNV [125]. These findings suggest that extended follow-up should be considered for CSCR patients, as this disease has a variable clinical course, especially those cases complicated with CNV. OCTA is thus a suitable tool for identifying potential patients at risk of developing a more complicated course of disease. In a study of 20 eyes with PCV secondary to chronic CSCR, OCTA could reveal the branching vascular network as a hyperflow lesion in both choriocapillaris and outer retina layers [126]. Corresponding to polypoidal dilation detected in ICGA, hyperflow round structure and hypoflow round structure were identified in 75% and 15% of cases, respectively. OCTA could be a useful tool to visualize the both branching vascular network and polypoidal lesions in CSCR complicated with PCV. Swept-source optical coherence tomography angiography was recently reported to have performance comparable to ICGA in detecting PCV [127].

#### 3.7.4. Limitations of Optical Coherence Tomography Angiography

Segmentation error and image artifacts are among the biggest challenges of OCTA application. When OCTA is used to assess active CSCR, SRF may cause shadowing effects that reduce the OCTA signal captured [128]; this type of artifact can be mitigated, though not completely eliminated, through using swept-source OCTA [129]. Various other imaging artifacts can also hinder accurate assessment. For example, motion artifact can produce horizontal dark lines or bands that are not apparent on OCT reflectivity maps. Moreover, the fast blood flow of choroidal vessels can create flow voids leading to fringe washout. In addition, decorrelation projection occurs when the OCTA signal from the retinal vasculature and new choroidal vessels above Bruch's membrane are seen together in the OCTA image within the choroid. Masking and unmasking artifacts can also be seen in regions of PED and atrophy [130]. Lastly, dark spots or areas are often seen in the OCTA images of CSCR eyes relating to several conditions, including PED, subretinal deposit, surrounding subretinal fibrin, choroidal cavitation, choroidal excavation, and choroidal fluid [131]. Given that the accurate interpretation of OCTA images requires the correct segmentation of tissue layers [132], training for OCTA assessors is important to ensure image quality and reduce errors [133].

Another drawback of OCTA is the lack of dynamic vascular information. OCTA cannot detect the leakage site of CSCR due to its inability to detect plasma flow [134]. Moreover, the leakage site is not directly associated with altered flow patterns in either the retinal vasculature or the choriocapillaris. PDT therefore cannot be applied solely based on OCTA imaging results [135]. In summary, each of the imaging techniques including FA, ICGA, and OCT has its own value, and they should be used in combination to achieve an accurate evaluation of CSCR eyes [136].

## 4. Conclusion

Advancements in imaging techniques and the combined application of several imaging techniques in a multimodal imaging approach have improved clinical understandings about the pathophysiology of CSCR, as well as improved the classification of the disease. The CSCR features provided by multimodal imaging tools offer better prognostication of the disease. As CSCR has wide range of clinical manifestations and disease courses, a thorough understanding of these imaging features can help clinicians formulate proper management plans for the disease.

## Data Availability

The data used to support the findings of this study are included within the article.
